# Editorial: Women in cardiovascular imaging

**DOI:** 10.3389/fcvm.2023.1249983

**Published:** 2023-08-02

**Authors:** Anna Vittoria Mattioli, Adelina Doltra, Claudia Prieto, Sabina Gallina

**Affiliations:** ^1^ Department of Medical and Surgical Sciences for Children and Adults, University of Modena and Reggio Emilia, Modena, Italy; ^2^ Non-invasive Imaging Section, Cardiovascular Institute Hospital Clínic, University of Barcelona, Barcelona, Spain; ^3^ King's College London, London, United Kingdom; ^4^ Pontificia Universidad Católica de Chile, Santiago, Chile; ^5^ Department of Neuroscience, Imaging and Clinical Sciences, G. D'Annunzio University, Chieti, Italy

**Keywords:** women, cardiovascular imaging, gender gap, education, prevention

**Editorial on the Research Topic** Women in cardiovascular imaging

To date there is still a great disparity between the sexes in the scientific field and this determines a small number of manuscripts that have a woman as principal investigator and corresponding author. At present, less than 30% of researchers worldwide are women (1). Long-standing biases and gender stereotypes are discouraging girls and women away from science-related fields, and in the area of STEM (Science, Technology, Engineering and Mathematics) research in particular. Analyzing the scientific literature and, specifically clinical trial, it emerges that the number of female Principal Investigators is substantially lower than males (2, 3).

The disparity between the sexes also emerges in careers. A meta-analysis on 218 studies found that men were 2.77 times more likely to be full professors (OR: 2.77, 95% CI: 2.57–2.98). Meta-regression by data collection year demonstrated improvement over time; however, subgroup analysis showed that gender disparities remain significant in the 2010–2020 decade (OR: 2.63, 95% CI: 2.48–2.80). The gender gap was present across all specialties and both within and outside of North America. Men published more papers with a mean difference of 17.2 (95% CI: 14.7–19.7) (3).

The proportion of female medical graduates appears to have increased over time, however, the gender ratio of physicians varies across specialties (4).

Today, women remain a minority in cardiology even though half of the medical school graduates are women over the last decade (49.3% in 2008 and 47.9% in 2018). Despite this, the proportion of women in cardiovascular disease fellowship training remains low (18% in 2008 and 23.4% in 2018), compared with other medical specialties (5).

This underrepresentation of women in cardiology also extends to women in leadership roles within the same community. The representation of women decreases progressively along the career path from medical student to cardiology fellow to practicing cardiologist and, finally, fewer women at leadership roles. The inclusion of women in the authorship guidelines is a good index of this underrepresentation (6, 7).

Science and gender equality are, however, essential to ensure sustainable development as highlighted by UNESCO (1). In order to change traditional mindsets, gender equality must be promoted, stereotypes defeated, and girls and women should be encouraged to pursue STEM careers. Achieving parity in leadership, peer mentorship, and role models can help motivate female medical students devote himself to STEM careers (8).

This special issue has collected 15 manuscripts of the highest quality written by women. The collection focuses on imaging technique from echocardiography, Computed Tomography-scan and Magnetic Resonance Imaging in their most advanced technical applications.

The article “Women physicians in cardiovascular magnetic resonance: Past, present, and future” summarizes the barriers that women in cardiovascular imaging have overcome over the past several years, the positive interventions that have been implemented to better support women in the field of cardiovascular magnetic resonance, and the challenges that still remain, with a special emphasis on women physicians (Sierra-Galan et al.).

Cardiovascular imaging specialty training is prolonged and demanding, consisting of medical school and residency (in either diagnostic radiology or internal medicine), followed by cardiology and dedicated cardiac imaging or cardiovascular fellowship. This long training discourages women from taking this path due to the difficulties in reconciling professional and personal activities above all.

Furthermore, given the well-known under-representation of women participants in cardiovascular studies, the increased recruitment of women demonstrated in trials with female leaders also have practical implications (9, 10).

The limited availability of gender-specific data in clinical research has significant implications for the quality of healthcare provided to women. Historically, medical research has focused predominantly on men, and findings from studies conducted primarily on male participants have been extrapolated to both genders. However, this approach neglects the fact that men and women can have distinct physiological, hormonal, and genetic differences that may influence disease risk, progression, and response to treatments (11, 12).

It is crucial to recognize the importance of including women in clinical trials and conducting adequate sex-specific analyses to gain a comprehensive understanding of women's health and to identify potential gender-specific effects of treatments and interventions (13).

Efforts to bridge the gender gap in medical research and healthcare can lead to more equitable and effective treatments for both women and men, ultimately improving overall health outcomes for all.

To improve the quality of care for women, researchers, healthcare providers, and policymakers should advocate for more inclusive clinical trial designs that prioritize gender representation. Additionally, conducting post-hoc subgroup analyses based on sex and presenting sex-specific results in research publications are essential steps toward advancing gender-specific healthcare.

Although clinical trials often include both sexes, there is often inadequate analysis of sex-based differences. It is important to develop subgroup analyzes and examine potential gender-specific effects within clinical trial data to identify any disparities or considerations unique to women (11, 12). Balancing study recruitment between men and women will help fill some gaps. Including an adequate number of women in clinical trials allows researchers to analyze and address sex-based differences in disease presentation, progression, and treatment response. It helps to identify any disparities or unique considerations that may affect women's health outcomes. Furthermore, the concept of personalized medicine, where medical decisions and treatments are tailored to an individual's specific characteristics, is gaining prominence. Understanding sex-based differences can lead to more tailored and effective healthcare approaches for both men and women (14, 15).

In addition, men and women may respond differently to medications due to variations in metabolism, hormone levels, and other biological factors. Having gender-specific data can help optimize drug dosing and minimize potential side effects in both sexes (16–18).

We believe the time has come for action and the scientific community must encourage women in STEM to write and position themselves as principal investigators.
